# Cell Penetrable Human scFv Specific to Middle Domain of Matrix Protein-1 Protects Mice from Lethal Influenza

**DOI:** 10.3390/v7010154

**Published:** 2015-01-14

**Authors:** Fonthip Dong-din-on, Thaweesak Songserm, Tippawan Pissawong, Potjanee Srimanote, Jeeraphong Thanongsaksrikul, Kanyarat Thueng-in, Pattra Moonjit, Preeda Lertwatcharasarakul, Watee Seesuay, Wanpen Chaicumpa

**Affiliations:** 1 Center for Agricultural Biotechnology, Kasetsart University, Kamphaeng Saen Campus, Nakhon Pathom 73140, Thailand; E-Mail: fddvt66@outlook.com; 2 Laboratory for Research and Technology Development, Department of Parasitology, and Center of Excellence on Therapeutic Proteins and Antibody Engineering, Faculty of Medicine Siriraj Hospital, Mahidol University, Bangkok 10700, Thailand; E-Mails: tpw029@gmail.com (T.P.); gskmu@hotmail.com (J.T.); mam_mt41@hotmail.com (K.T.); watee.see@gmail.com (W.S.); 3 Department of Veterinary Pathology, Faculty of Veterinary Medicine, Kasetsart University, Kamphaeng Saen Campus, Nakhon Pathom 73140, Thailand; E-Mails: fvettss@ku.ac.th (T.S.); fvetpas@ku.ac.th (P.M.); preeda_l@ku.ac.th (P.L.); 4 Chulabhorn International College of Medicine, Thammasat University, Pathumthani 12120, Thailand; 5 Graduate Program in Biomedical Science, Faculty of Allied Health Sciences, Thammasat University, Pathumthani 12120, Thailand; E-Mail: psrimanote01@yahoo.com.au; 6 Department of Microbiology and Immunology, Faculty of Veterinary Medicine, Kasetsart University, Bangkok 10900, Thailand

**Keywords:** Cell penetrating antibody, H5N1, human ScFv, influenza, influenza virus, matrix protein-1 (M1), middle domain of M1, molecular docking, phage display, transbody

## Abstract

A new anti-influenza remedy that can tolerate the virus antigenic variation is needed. Influenza virus matrix protein-1 (M1) is highly conserved and pivotal for the virus replication cycle: virus uncoating, assembly and budding. An agent that blocks the M1 functions should be an effective anti-influenza agent. In this study, human scFv that bound to recombinant M1 middle domain (MD) and native M1 of A/H5N1 was produced. Phage mimotope search and computerized molecular docking revealed that the scFv bound to the MD conformational epitope formed by juxtaposed helices 7 and 9 of the M1. The scFv was linked molecularly to a cell penetrable peptide, penetratin (PEN). The PEN-scFv (transbody), when used to treat the cells pre-infected with the heterologous clade/subclade A/H5N1 reduced the viral mRNA intracellularly and in the cell culture fluids. The transbody mitigated symptom severity and lung histopathology of the H5N1 infected mice and caused reduction of virus antigen in the tissues as well as extricated the animals from the lethal challenge in a dose dependent manner. The transbody specific to the M1 MD, either alone or in combination with the cognate human scFvs specific to other influenza virus proteins, should be an effective, safe and mutation tolerable anti-influenza agent.

## 1. Introduction

Type A influenza viruses are etiologic agents of highly contagious acute respiratory disease, *i.e.*, influenza, of humans, other mammals and avian species [1]. The world has experienced several catastrophic influenza pandemics/panzootics which have resulted in exceptionally high fatality rates among infected humans and animals, especially poultry, which has led to severe economic loss [2,3,4]. Vaccination is the most effective measure for influenza intervention, both prevention of the infection and mitigation of the symptom severity. However, there are limitations in the production and efficacy of the current influenza vaccines which the vaccine viruses have to be propagated in embryonated eggs [5,6]. New generation vaccines that are egg-independent, rapidly and timely produced at the time of high demand, more tolerable to virus antigenic variations and cross-protect against viruses of different strains and subtypes, such as those based on the highly conserved influenza virus proteins, have been sought [7,8]. For treatment of influenza, only two families of anti-viral drugs have been approved for clinical use, namely, inhibitors of the M2 ion channel activity and the neuraminidase inhibitors. Drug resistant influenza virus variants have continuously emerged causing a failure of influenza treatment and consequently, a frequent case fatality especially among small children, elderly and immunocompromised subjects [9,10]. There is a perceived necessity of developing a new and effective remedy for influenza treatment.

Influenza A virus has a negative sense, single-stranded RNA genome which encodes 18 different functional proteins (PB2, PB1, PB1-F2, PB1-N40, PA, PA-X, PA-N155, PA-N182, HA, NP, NA, M1, M2, M42, NS1, NEP, NS3 and NEG8/NSP/NS4) [11]. Among them, the predominant matrix protein-1 (M1) which lies underneath the viral envelope is predominant and highly conserved across the influenza A subtypes [12]. This protein is pivotal for the virus infectious cycle as it involves in many steps of the replication process. M1 initiates the virus uncoating by freeing the viral ribonucleoprotein (vRNP) from endosome into cytoplasm for further replication in the nucleus [13]. M1 synergizes the virus nuclear export protein (NEP) in transporting the newly synthesized vRNPs to the cytoplasm for further assembling [14,15,16]. The protein also mediates self-oligomerization which forms a meshwork that supports other viral components in the new particle morphogenesis [17,18,19,20,21]. Each M1 molecule is composed of three functionally different domains including N-terminal (ND, residues 1–67), middle (MD, residues 91–158) and C-terminal (CD, residues 165–252) [22]. Among the three domains, MD is the most attractive target of a new anti-influenza agent as it involves in several M1 functions [20,23]. In this study, engineered cell penetrable human monoclonal single chain antibody (human scFv) that bound specifically to the MD of influenza A virus subtype H5N1 (A/H5N1) were produced. Ability of the MD specific transbody to interfere with the virus replication and pathogenesis were evaluated.

## 2. Material and Methods

### 2.1. Influenza A Viruses

Influenza viruses used in this study were A/duck/Thailand/144/2005 (H5N1), clade 1 [24] and mouse adapted A/chicken/Thailand/NP-172/2006 (H5N1), clade 2, subclade 3 (DQ 999871-8). They were propagated in 8–10 day old specific pathogen-free embryonated eggs.

### 2.2. Preparation of Native M1

MDCK cells infected with mouse adapted, A/chicken/Thailand/NP172/2006 at MOI 1.0 were cultured in complete Dulbecco’s modified Eagle’s medium [DMEM (Invitrogen, Carlsbad, CA, USA) supplemented with 10% heat-inactivated fetal bovine serum (HyClone, Northumberland, UK), 2 mM L-glutamine, streptomycin (100 µg/mL) and penicillin (100 units/mL)] for 24 h before subjecting to sonication in phosphate buffered saline, pH 7.4 (PBS) on ice. The homogenate was centrifuged (12,000× *g*, 4 °C, 20 min) and the supernatant (cell lysate) containing the native M1 protein (nM1) was collected. CNBr-activated Sepharose™ 4B (GE Healthcare, Rapsgatan, Uppsala, Sweden) coupling with rabbit polyclonal immunoglubulin to M1 was used for native M1 purification. The bead was incubated with the cell lysate containing nM1 on ice for 2 h, washed and the protein bound to the affinity beads was eluted with 0.1 M glycine buffer, pH 3.0. The eluted fraction containing nM1 (determined by SDS-PAGE and protein staining) was neutralized by using 1 M Tris-HCl, pH 9.0.

### 2.3. Recombinant MD (rMD) Preparation

Total RNA was extracted from the A/duck/Thailand/144/2005(H5N1) virus by using TRIzol reagent (Invitrogen). Complementary DNA (cDNA) was synthesized from the RNA by using Revert Aid™ H Minus Strand cDNA synthesis kit (Fermentas, Vilnius, Lithuania). The DNA was PCR amplified by using nucleotide primers designed from the M1 gene sequence of the database (accession no. AY626114): forward: 5'-GGA TCC GAA TGG AAA TGG AGA T-3' and reverse: 5'-AAG CTT CTG TCT GTG AGA CCG-3'. *BamH*I and *Hind*III endonuclease restriction sites (underlined) were included at the 5' ends of both primers. The PCR thermal cycles were: initial denaturation at 94 °C for 5 min, 30 cycles of 94 °C for 1 min, 50 °C for 1 min, and 72 °C for 1 min, and 72 °C for 10 min. The PCR product was ligated into the pGEM^®^-T Easy vector (Promega, WI, USA) and the recombinant vector was put into JM109 *E. coli*. The DNA insert was verified by nucleotide sequencing. The MD coding sequence was subcloned into pET20b(+) expression vector (Novagen, Medison, WI, USA) and the vector was introduced into BL21 (DE3) *E. coli*. A selected transformed bacterial colony was grown under 0.5 mM IPTG (USB, OH, USA) induction and the rMD was purified from the bacterial lysate by using Ni-NTA agarose (Invitrogen). 

### 2.4. Bio-Panning for Selection of Phage Clones that Bound to rMD and Production of the MD Specific-human scFv

A human scFv phage display library was constructed previously [25].The antibody diversity of this library was approximately 2.6 × 10^8^. After propagating in TG1 *E. coli* and co-infecting the bacteria with M13KO7 helper phages, approximately 6 × 10^12^ cfu/mL of complete phage particles were obtained. Phage clones that bound to the rMD were selected from the library that had been subtracted with lysate of BL21 (DE3) *E. coli* carrying pET20b(+). An ELISA well was coated with 10 µg of rMD in 100 µL of 0.05 M Na_2_CO_3_, pH 9.6 (coating buffer). After washing the well with PBST and blocked with 3% BSA (Sigma-Aldrich, Saint Louis, MI, USA) in PBS, the subtracted phage library (~3 × 10^11^ particles) was added into the antigen-coated well and the plate was incubated at 37 °C for 1 h. Unbound phages were removed by washing with PBST and an aliquot of a log phase grown HB2151 *E. coli* culture was added to the well. Phage transfection was allowed to occur at 37 °C for 1 h; the preparation was spread onto 2× YT agar containing 100 µg/mL amplicillin and 2% glucose (2× YT-AG) and incubated at 37 °C for 16 h. The phage-transformed HB2151 *E. coli* colonies appeared on the agar plate were PCR screened for the human scFv coding sequences (*huscfv*) by using the pCANTAB5E specific primers, *i.e.*, forward (R1): 5'-CCA TGA TTA CGC CAA GCT TT-3' and reverse (R2): 5'-GCT AGA TTT CAA AAC AGC ACA AAG G-3') [25]. The expected size of the *huscfv* amplicon was ~1000 bp. The *E. coli* carrying *huscfv*-phagemids were screened for their ability to produce human scFv. Individual colonies were grown under 0.5 mM IPTG induction for 3 h; the bacterial cells were harvested, homogenized and centrifuged at 10,000× *g* at 4 °C for 15 min. Supernatants were checked for the presence of the scFv by Western blotting. Each *E. coli* lysate was subjected to 12% SDS-PAGE and the gel-separated components were blotted onto an NC. The NC was blocked with 3% skim milk in PBS before incubating with mouse monoclonal anti-E tag (Abcam, Cambridge, UK). The human scFv-anti-E tag reactive bands were visualized by using goat anti-mouse immunoglobulin-alkaline phosphatase (AP) conjugate (Southern Biotech, Birmingham, AL, USA) and BCIP/NBT substrate (KPL, Gaithersburg, MD, USA). The scFvs were purified by using DEAE anion exchange column chromatography. The amounts of the scFvs in the column flow-through fluids were standardized densitometrically.

### 2.5. Characterization of the Human scFvs

Antigenic specificity of the human scFvs from individual HB2151 *E. coli* lysates was determined by indirect ELISA and Western blot analysis. Purified Native M1 and rMD (1 µg in 100 µL coating buffer, respectively) were added to wells of an ELISA plate (Corning, New York, USA). Well coated with BSA served as control antigen. After incubating at 37 °C for 16 h, the unbounded proteins were removed by washing with PBST and the well surface was blocked with 3% skim milk in PBS. After washing, 100 µL of the human scFv preparations were added appropriately and incubated at 25 °C for 1 h. Lysate of original HB2151 *E. coli* was used as control negative scFv. After washing, mouse monoclonal anti-E tag antibody diluted 1:3000 (100 µL) was added to each well and incubated at 37 °C for 1 h. Goat anti-mouse immunoglobulin-horseradish peroxidase (HRP) conjugate (Southern Biotech) (100 µL of 1:3000) and ABTS substrate (KPL) were used for color development. OD_405nm_ of the content in each well was determined against blank (well to which PBS was added instead of the scFv or HB2151 *E. coli* lysate). The *E. coli* clones which their expressed scFvs gave the OD at least two times higher than the BSA control were selected and the scFvs were subjected to Western blot analysis for confirmation of their binding to the native M1 and rMD. Briefly, purified native M1 and rMD were subjected to 14% SDS-PAGE; the separated components were blotted onto an NC and the blotted NC was cut vertically into strips. The NC strips were blocked with 3% skim milk in PBS before incubating individually with the scFv preparations at 25 °C for 1 h. Lysate of HB2151 *E. coli* was used as negative antibody control. The antigen-antibody reactive bands at the expected sizes (~26 kDa for native M1 and ~14 kDa for rMD) were revealed by probing the NC with mouse monoclonal anti-E tag, goat anti-mouse immunoglobulin-AP and BCIP/NBT substrate. Restriction fragment length polymorphism (RFLP) of the *huscfv* sequences in individual *huscfv*-phagemid transformed *E. coli* clones was determined as described previously [25]. The *huscfv*s were also sequenced and the deduced. Complementarity determining regions (CDRs) and immunoglobulin framework regions (FRs) of the VH and the VL domains of the scFv amino acid sequences were predicted by using the International Immunogenetics information system (IMGT/VQUEST).

### 2.6. Large Scale Production of the Human scFv

The *huscfvs* of *E. coli* clones of interest were subcloned into pET23b(+) (Novagen) *via Hin*dIII and *Not*I restriction sites and the recombinant plasmid was introduced to into BL21 (DE3) *E. coli*. After verification of the DNA inserts by plasmid sequencing, appropriate BL21 (DE3) *E. coli* colonies were grown under 0.5 mM IPTG induction for 3 h. The scFvs were purified from the *E. coli* lysates by using Ni-NTA agarose (Invitrogen). Each antibody preparation was dialyzed against PBS and maintained at −20 °C until use.

### 2.7. Cell Penetrable Human scFv Specific to MD of M1 

In order to produce cell penetrable human scFv, a previously constructed recombinant plasmid backbone, *i.e.*, *penetratin* (*pen*)-pET23b(+) was used [26]. Penetratin (PEN) is a 16 amino acid cell penetrating peptide which can carry the cargo protein across a eukaryotic cell membrane without causing membrane damage. The *huscfv* sequence from the recombinant pCANTAB5E phagemid was inserted into the *pen*-pET23b(+) plasmid construct downstream of the *pen* sequence *via* the *Sfi*I and the *Not*I endonuclease restriction sites. The recombinant plasmid was introduced to into BL21 (DE3) *E. coli* and the *huscfv* insert was verified by DNA sequencing. Appropriate *E. coli* colony was grown under 0.5 mM IPTG induction and the PEN-scFv was purified from the *E. coli* lysate by using Ni-NTA agarose (Invitrogen).

### 2.8. Cell Internalization of PEN-scFv

Cell penetrating ability of the PEN-scFv was determined as described previously [27]. MDCK cells were added to glass coverslips placed in the wells of a 24-well tissue culture plate (5 × 10^4^ cells/well). The cells were grown in complete DMEM at 37 °C in a 5% CO_2_ incubator for 24 h. After rinsing twice with PBS, 20 µg of PEN-scFv was added for 1 h; the cells were washed with PBS, fixed with 4% paraformaldehyde, washed again, and permeated with 0.2% Triton X-100 for 15 min. After washing, the cells were blocked with 3% BSA in PBS. The PEN-scFvs inside the cells were detected by probing with mouse monoclonal anti-6× histidine tag (Abcam) dilute 1: 3000 and Alexa Flour 488-labeled chicken anti-mouse immunoglobulins (Invitrogen) diluted 1:500, respectively, with washing between the steps. DAPI (Invitrogen) was used for localization of nuclei. The stained cells were subjected to 0.5 µm laser sectional confocal microscopy to locate PEN-scFvs at different cellular layers.

### 2.9. Interference of the Influenza Virus Replication by MD Specific-PEN-scFv

MDCK cells were added to wells in a 24-well tissue culture plate (5 × 10^4^ cells/well) and grown in complete DMEM to 80% confluent monolayer. They were then infected with A/chicken/Thailand/NP-172/2006 (H5N1) at MOI 0.1 and incubated at 37 °C for 2 h. The cells were washed with plain DMEM. Two µg each of the MD specific-scFv/PEN-scFv, irrelevant (control) human scFv/PEN-scFv (specific to puffer tetradotoxin) [28], diluted lysate of pET23b(+) transformed BL21 (DE3) *E. coli* (non-antibody control), mouse polyclonal antibodies (PAb) to M1, PAb to H5N1, rimantadine (0.5 µg) and diluent control were added appropriately to triplicate wells of the infected cells. The plates were incubated at 37 °C in 5% CO_2_ incubator for 15 h. H5N1 M1 mRNA in all culture fluids and inside the cells were determined by quantitative real-time RT PCR (qPCR) [29]. Three independent experiments were done.

### 2.10. Therapeutic Efficacy of scFv/PEN-scFv in Influenza Virus Infected Mice

All *in vivo* studies were conducted under the guidelines of the National Research Council of Thailand and were approved by the Ethical Committee of the Faculty of Veterinary Medicine, Kasetsart University, Thailand. Female BALB/c mice (4–6 weeks old) were from the National Laboratory Animal Center, Mahidol University, Thailand. They were maintained at the animal facilities of the Faculty of Veterinary Medicine, Kasetsart University, Thailand. Feedstuff and water were provided *ad libitum*. Twelve groups of 10 mice were set up. Groups 1–11 were infected intranasally with 10 MLD_50_ of mouse adapted A/chicken/Thailand/NP-172/2006(H5N1) (20 µL; 10 µL into each nostril) while the group 12 mice served as non-infected control and received PBS intranasally. Twelve hours post-infection (p.i.), the infected mice of groups 1 and 2 and 3 and 4 were treated intraperitoneally (i.p.) with the 5 and 10 mg/kg body weight of MD specific scFv and PEN-scFv, respectively. The mice of groups 5–11 were treated i.p. with 10 mg/kg of control scFv, control PEN-scFv [28], lysate of pET23b(+) transformed BL21 (DE3) *E. coli*, mouse PAb to M1, mouse PAb to H5N1 and rimantadine and PBS, respectively. The respective treatments were repeated at 24, 48, 72 and 96 h p.i. Non-infected mice (group 12) were injected individually with PBS. Four mice of each group were sacrificed at 96 h p.i. and their left lungs were collected in 10% buffered formalin for histopathological study and immunohistochemical staining while the right lungs were kept in RNA Later (Qiagen, Valencia, CA, USA) for determining the viral loads by qPCR. The remaining mice were observed daily and the numbers of dead and alive mice were recorded. The experiments were terminated on day 21 p.i. The results of the three reproducible experiments were combined and reported herein. 

### 2.11. Quantitative Real-Time RT PCR (qPCR)

For qPCR, the vRNA was extracted from 50 mg of each lung sample kept in the RNA Later by using viral nucleic acid extraction kit II (Geneaid, New Taipei City, Taiwan). Amount of the RNA in each preparation was determined by using NanoDrop ND-1000 Spectrophotometer (Thermo Scientific). One-step Brilliant II SYBR^®^ green qRT-PCR master mix kit (Agilent Technologies, Santa Clara, CA, USA) and primers to influenza A virus M1, *i.e.*, forward 5'-CTT CTA ACC GAG GTC GAA TCG TA-3' and reverse 5'-TCC ATG AGA GCC TCG AGA T-3' were used. PCR master mix (12.5 μL) containing 200 ng of RNA in 4.75 µL of DEPC-water, 6.25 µL of 2× Brilliant II SYBR green qRT-PCR master mix buffer, 0.5 µL of RT/RNase block enzyme and 200 nM of each primer (0.5 µL) was prepared on ice. The PCR was carried out using Mx 3000PTM instrument (Stratagene, San Diego, CA, USA) and the condition was: reverse transcription at 42 °C for 1 h, initial denaturation at 95 °C for 10 min, 40 PCR cycles at 95 °C for 30 s, 58 °C for 30 s and 72 °C for 30 s. To analyze the dissociation curve, the following thermal profile was determined: 95 °C for 1 min and ramped down to 55 °C (0.5 °C/s) and then ramped up to 95 °C. A standard curve was constructed from Ct of ten-fold dilutions of the pET20b(+) carrying M1 sequence (1 ng to 1000 ng or 2.07 × 10^8^ to 2.07 × 10^11^ copies). Ct (log_10_ of the RNA copies) of each sample was calculated from the standard curve.

### 2.12. Viral Foci Assay

For measurement of viral titers, 50 mg of each lung sample kept in 1 mL of viral transport medium was homogenized by tissue blender (Tissue Tearor™ homogenizer Biospec, Bartlesville, OK, USA). The preparations were centrifuged at 10,000× *g*, 4 °C for 15 min; the supernatants were filtered through 0.2 µm membranes. Each sample was added to MDCK monolayer established in a 24-well tissue culture plate wells (5 × 10^4^ cells/well) and kept at 37 °C in a 5% CO_2_ incubator for 2 h. The fluids in all wells were discarded and the cells were rinsed twice with plain DMEM before replenishing with DMEM supplemented with 2% heat-inactivated fetal bovine serum (HyClone), 2 mM L-glutamine, streptomycin (100 µg/mL) and penicillin (100 units/mL) and kept for 15 h. After rinsing twice with PBS, the cells were fixed with 4% paraformaldehyde, washed, permeated with 0.2% Triton X-100, washed with PBST and blocked with 3% BSA in PBS. Mouse monoclonal antibody against-NP (produced by injecting three doses of recombinant NP mixed with alum intramuscularly into mice at two week intervals) was added to the cells and kept at 37 °C for 1 h. Goat anti-mouse immunoglobulin-alkaline phosphatase (AP) conjugate (Southern Biotech) and BCIP/NBT substrate (KPL) were used for chemiluminescent development. The reaction was stopped by rinsing the cells with distilled water. The preparations were examined under a light microscope.

### 2.13. Histopathology of the Mouse Lungs

For histopathological study, 5 µm sections of the mouse lung samples that had been fixed in 10% neutral buffered formalin and embedded in paraffin were prepared. The tissue sections were stained with hematoxylin and eosin (H & E) dyes and observed under a light microscope. Lung sections of a normal mouse served as control.

### 2.14. Immunohistochemical Staining of the Virus Antigen in the Mouse Lungs

For detection of the influenza virus antigen (nucleoprotein, NP), 5 µm paraffin sections of the fixed mouse lung samples were deparaffinized with xylene before rehydrating with a decreasing gradients of ethanol, *i.e.*, absolute, 95%, 80% and 70%, respectively. Endogenous peroxidase was quenched by using 3% hydrogen peroxide in PBS. After thorough washing, 200 µg of proteinase-K (Sigma-Aldrich) in PBS was applied to each tissue section and kept at 37 °C, 20 min for the antigen retrieval. Non-specific binding was blocked by flooding each slide with 100 µL of 2% BSA (Sigma-Aldrich) in PBS. Mouse monoclonal anti-NP was applied onto the tissue sections and kept at 37 °C for 1 h. Goat anti-mouse immunoglobulin-HRP conjugate (Southern Biotech) and freshly prepared 3, 3'-diaminobenzidine (DAB, BioBasic, Amherst, NY, USA) containing 3% of hydrogen peroxide were used for color development. The reaction was stopped by rinsing the slides with distilled water. Finally, the slides were counter-stained with hematoxylin, mounted and examined under a light microscope (original magnification 40×).

### 2.15. A Search for Phage Mimotopic Peptides for Determining the scFv Presumptive Epitopes

Ph.D. 12™ phage display peptide library (New England Biolabs, Ipswich, MA, USA) was used to determine the phage peptides that bound to the scFv [27]. Briefly, MD specific-scFv (10 µg) was coated onto well surface of an ELISA plate (Corning). After blocking with 5% skim milk, 100 µL of the peptide phage library (10^11^ pfu) were added to a well and the plate was incubated at 37 °C for 1 h. Unbound phages were removed and the well was washed before adding with 150 µL of mid log phase grown ER2738 *E. coli*. After keeping the plate at 37 °C for 20 min, the phage transfected *E. coli* were grown in LB broth at 37 °C for 5 h. Propagated phages were collected by precipitating the bacterial culture supernatant with PEG-NaCl and used for the next panning round. The panning was repeated three times. Phages recovered from the *E. coli* culture supernatant of the third panning round were spread on an agarose topped-LB plate containing X-Gal and IPTG. Ten blue plaques were picked randomly and grew in LB broth for 6 h. The phage DNAs were extracted from the culture supernatants and sequenced. The peptide sequences were deduced from the phage DNA by DNAMAN software version 4.15. The phage mimotopic peptides were classified into mimotope types by using Phylogeny ClustalW. The sequence of each mimotope type was multiply aligned with the M1 linear sequence (accession no. AY626114), using Kalign for identification of the M1 residues bound by the scFv (the presumptive scFv epitope).

### 2.16. Competitive ELISA for Verification of the Phage Mimotopes

Phage clones displaying the mimotopic peptides that bound to the scFv were tested by competitive ELISA for determining their capacity in blocking the scFv binding to the rMD as described previously [29]. Mimotopic phages were propagated in ER2738 *E. coli* and the titers of the amplified phages were determined according to manufacturer’s instruction (New England Biolabs). Various amounts of the mimotopic phages or their mixture (10^4^, 10^5^, 10^6^ pfu) in 50 µL were incubated individually with fixed amount of scFv (5 µg in 50 µL) at 37 °C for 1 h. The scFv mixed with control peptide phages (did not bind to the scFv) served as background inhibition control while the scFv in buffer was negative inhibition control (maximum binding, 100%). After adding individual mixtures to the rMD coated wells and incubated at 37 °C for 1 h, all wells were washed and mouse monoclonal anti-E tag antibody were added, followed by goat anti-mouse immunoglobulin-HRP conjugate and ABTS substrate, respectively, with washing between the steps. The plate was kept in darkness for 30 min. OD_405nm_ of the fluid in each well was measured against blank (rMD coated well added with mouse monoclonal anti-E tag antibody, goat anti-mouse immunoglobulin-HRP conjugate and ABTS substrate). The % ELISA inhibition of the scFv binding to the immobilized antigen by individual phage mimotopic types or their mixture was calculated. The % ELISA inhibition = (OD of maximum binding–OD of test)/(OD of maximum binding) × 100.

### 2.17. Computerized Procedure for Determining the Interaction between the Human scFv and M1 MD of Influenza A Virus

Computerized procedure was used for determining the interaction between the scFv and the M1 MD of influenza A virus. The amino acid sequence of the antibody and the MD were subjected to homology modeling by the I-TASSER server service (http://zhanglab.ccmb.med.umic.edu/I-TASSER/) [30,31]. The qualities of the I-TASSER predicted 3D structural models were subsequently refined in order to make them become near to their native state by using the high-resolution protein structure refinement, ModRefiner (http://zhanglab.ccmb.med.umich.edu/ModRefiner/) [32] and the fragment-guided molecular dynamics (FG-MD) simulation (http://zhanglab.ccmb.med.umich.edu/FG-MD/) [33].The refined models were docked according to the Fast Fourier Transform (FFT)-based program, *i.e.*, PIPER. The antibody mode available on the automated ClusPro 2.0 protein-protein docking server was used for the antibody-protein docking [34,35,36,37,38]. The largest cluster size with a minimal local energy and a near native state of the protein conformation was chosen. The Pymol software [39] was used for building and visualizing the molecular interactions of the structural models.

### 2.18. Statistical Analysis

Comparisons between the results of the tests and the controls were performed using unpaired *t*-test. *p* < 0.05 was considered statistically significant.

## 3. Results

### 3.1. Production of Recombinant M1 Middle Domain (rMD)

**Figure 1:**
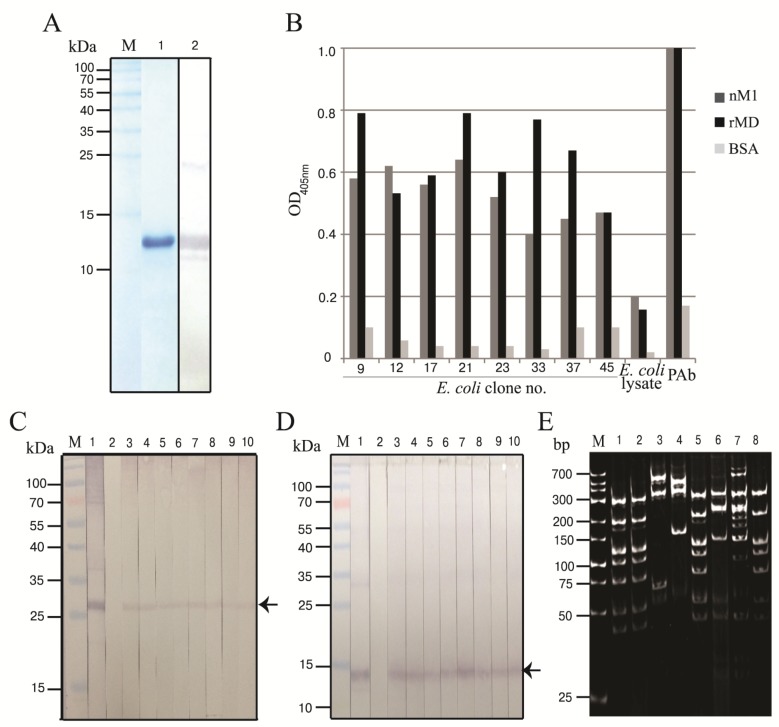
Recombinant M1 middle domain (rMD) and characterization of the selected phagemid transformed-HB2151 *E. coli* clones. (**A**) Recombinant MD. Lane M, protein molecular mass marker; lane 1, SDS-PAGE separated-rMD stained with Coomassie™ Brilliant Blue G-250 dye; lane 2, Western blot pattern of the purified rMD revealed by mouse monoclonal anti-6× histidine tag, goat anti-mouse immunoglobulin-alkaline phosphatase and BCIP/NBT substrate; (**B**) Human scFvs from eight phagemid transformed HB2151 *E. coli* clones (nos. 9, 12, 17, 21, 23, 33, 37 and 45) showed higher ELISA signals to native M1 and rMD than to the BSA (control antigen). Mouse polyclonal antibody to rM1 (PAb) and normal *E. coli* lysate were used as a positive and negative antibody controls, respectively; (**C**,**D**) Western blot results for determining the binding of the scFvs of the eight clones to nM1 and rMD, respectively (lanes 3–10). Lanes M, protein molecular mass marker; lanes 1 and 2, PAb to rM1 and *E. coli* lysate which served as positive and negative antibody controls, respectively; (**E**) DNA banding patterns (RFLP) of the *huscfv* sequences of the eight transformed HB2151 *E. coli* clones after *Mva*I digestion, 12% acrylamide gel electrophoresis and ethidium bromide staining. Lane M, low molecular weight DNA ladder.

DNA sequence coding for recombinant middle domain of matrix protein-1 (rMD) of A/duck/Thailand/144/2005 (H5N1, clade 1) was successfully amplified, cloned and put into BL21 (DE3) *E. coli*. The SDS-PAGE-separated pattern of the rMD expressed by the transformed *E. coli* stained with Coomassie™ Brilliant Blue G-250 dye (CBB) is shown in Figure 1A. The MD deduced amino acid sequence showed 100% homology to the MD sequence of H5N1 isolate (accession no. AY626114) used for designing the MD specific primers and also the various other H5N1 strains (data not shown).

### 3.2. Human scFvs Specific to nM1 and rMD

The purified rMD was used as an antigen in phage bio-panning for selecting the rMD bound phage clones from a human scFv phage display library. The antigen bound phages were put into HB2151 *E. coli* and the bacteria were grown on a selective agar plate. Fifty phage-transfected *E. coli* clones were picked randomly from the agar plate and screened for the presence of human scFv coding sequences (*huscfvs*) by PCR from which 19 clones (38%) were positive. Among them, eight clones expressed the scFvs (nos. 9, 12, 17, 21, 23, 33, 37 and 45) as determined by Western blotting. Specific binding of the scFvs of the eight *E. coli* clones to native and recombinant M1 proteins was determined by indirect ELISA using BSA as control antigen. The scFvs from all *E. coli* clones bound to the antigens and gave significant ELISA OD_405nm_ above the BSA control (Figure 1B). Western blot analysis verified the indirect ELISA results (Figure 1C,D). The *huscfv* DNAs of the eight clones revealed seven different DNA banding patterns after cutting by *Mva*I restriction endonuclease and 14% SDS-PAGE and ethidium bromide staining (Figure 1E). Clone nos. 9, 12, 33 and 45 had the same DNA pattern while clone nos. 17, 21 and 37 showed different patterns.

### 3.3. Cell Penetrable Human scFv and the Cellular Entry

**Figure 2:**
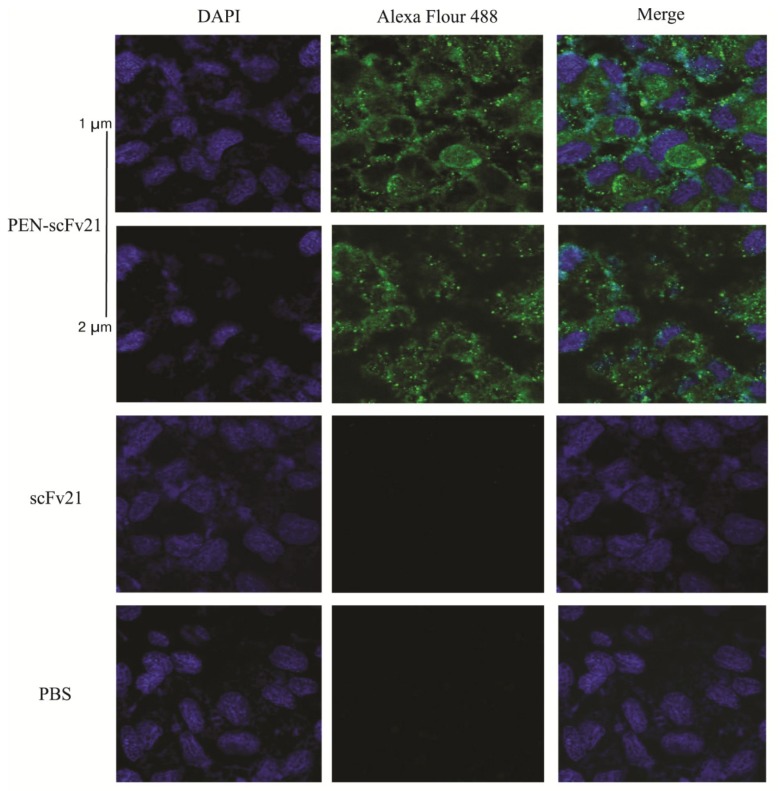
Intracellular localization of the penetratin (PEN)-scFv21 in MDCK cells revealed by laser sectional fluorescent confocal microscopy. The two upper rows, sections of MDCK cells (1 and 2 µm from the surface, respectively) incubated with PEN-scFv21; the third and the bottom rows, cells incubated with scFv21 and PBS, respectively. Left, middle and right panels of all rows, the cells were stained with DAPI for nuclei (blue), mouse monoclonal anti-6 × histidine tag and Alexa Flour 488 for PEN-scFv21 (green fluorescence) and merge, respectively.

The *huscfv* sequence of clone no. 21 (scFv of this clone gave high ELISA OD_405nm_ against the nM1 and the rMD and also preliminary neutralizing experiment indicated that the scFv21 was the best in reducing the virus released from the treated infected cells and inside the cells) was subcloned successfully from pCANTAB5E phagemid into *pen*-pET23b(+) vector [26]. The scFv21 and its cell penetrable version, *i.e.*, PEN-scFv21, were expressed and purified from the transformed BL21 (DE3) *E. coli* clones carrying the respective vectors grown under IPTG induction. After incubating the MDCK cells with 20 µg of the PEN-scFv21 for 1 h, the PEN-scFv21 was found to localize inside the treated MDCK cells (Figure 2).

### 3.4. Inhibition of Influenza Virus Replication in MDCK Cells by MD Specific-scFv and PEN-scFv

**Figure 3:**
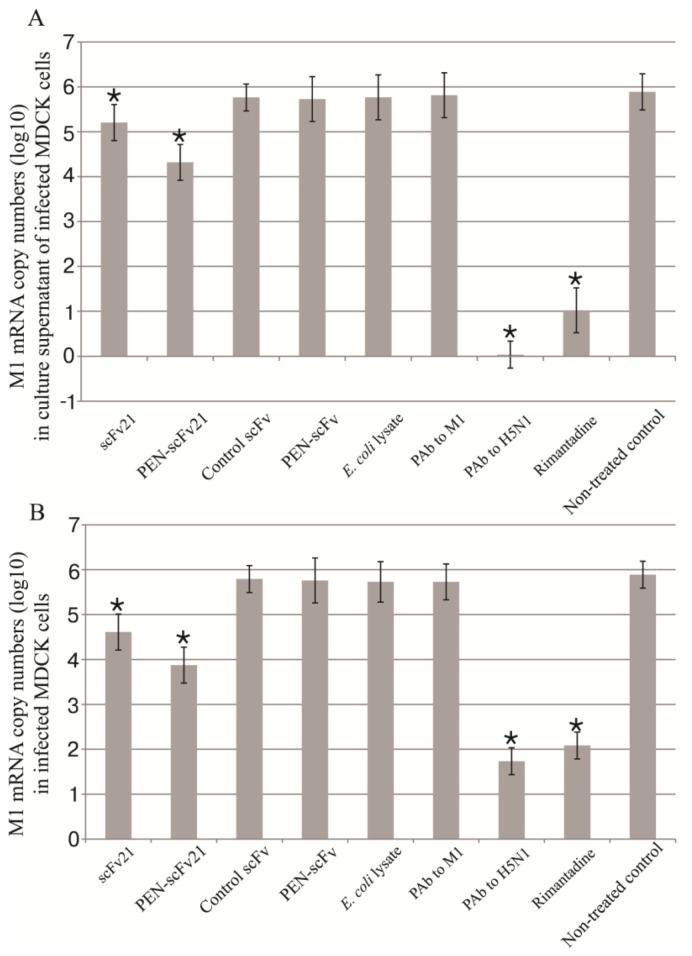
M1 mRNA copy numbers (log_10_) in (**A**) culture supernatants and (**B**) inside the cells of adamantane sensitive A/chicken/Thailand/NP-172/2006 (H5N1) infected MDCK cells that had been treated with scFv21, PEN-scFv21, control scFv, control PEN-scFv, *E. coli* lysate, polyclonal antibody (PAb) to M1, PAb to H5N1 and rimantadine in comparison with the non-treated infected cell control.*, significant difference from the non-treated infected control (*p* < 0.05).

MDCK cells were infected with 0.1 MOI of A/chicken/Thailand/NP-172/2006 (H5N1) (mouse adapted, adamantane-sensitive virus, clade 2, subclade 3). The infected cells were treated with MD specific scFv21, PEN-scFv21, control scFv, control PEN-scFv, lysate of original BL21 (DE3) *E. coli*, polyclonal antibody (PAb) to M1, PAb to H5N1 and rimantadine. The infected cells in culture medium alone served as non-treated infected control. After 18 h, M1 mRNA in culture supernatants and inside the cells of all treatment groups were determined by qPCR (Figure 3A,B, respectively). There were significant reduction of viral mRNA in culture supernatants and inside the infected MDCK cells treated with scFv21, PEN-scFv21, PAb to H5N1 and rimantadine compared with the non-treated infected control (*p* < 0.01). The infected cells treated with irrelevant scFv, irrelevant PEN-scFv, original *E. coli* lysate and PAb to M1 had negligible reduction of the viral mRNA in both cell spent medium and inside the cells compared to the non-treated infected cells (*p* > 0.05). The viral mRNA copy numbers in both culture supernatants and inside the cells were: PAb to H5N1 < rimantadine < PEN-scFv21 < scFv21 < irrelevant scFv = irrelevant PEN-scFv = *E. coli* lysate = PAb to M1 = culture medium alone.

### 3.5. Therapeutic Efficacy of MD Specific-scFv and PEN-scFv in A/H5N1 Infected Mice

**Figure 4:**
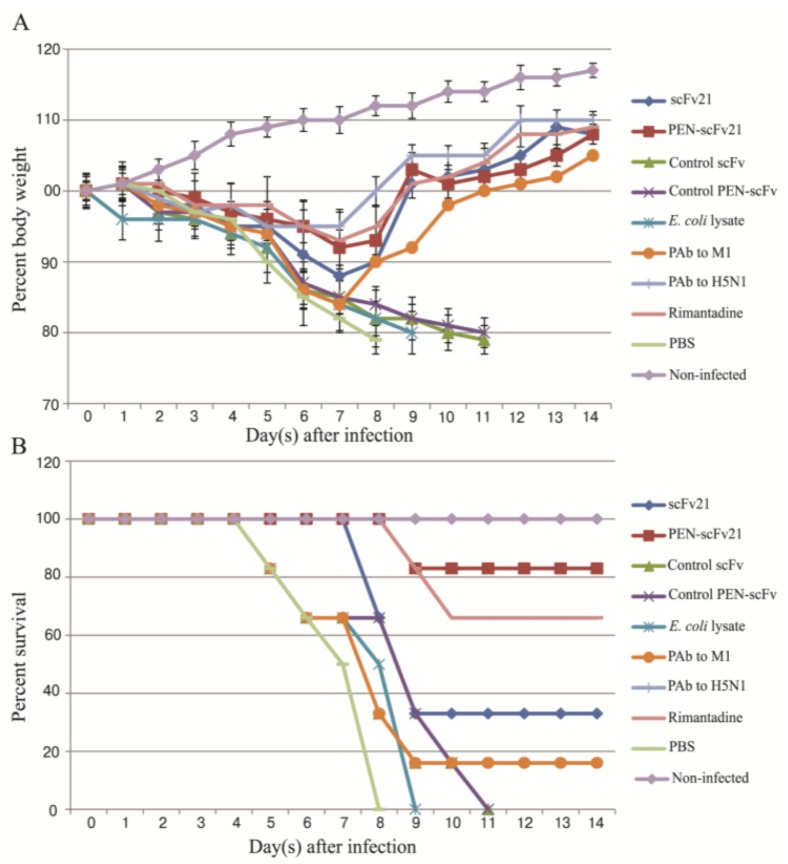
Percent body weight (mean and SD) and survival of infected mice that received different treatments, *i.e.*, scFv21, PEN-scFv21, control scFv, control PEN-scFv, *E. coli* lysate, PAb to M1, PAb to H5N1, rimantadine and PBS in comparison with the non-infected mice. (**A**) Percent body weight; (**B**) Percent survival.

Morbidity (body weight loss) and percent survival of mice infected with 10 MLD_50_ of mouse adapted A/chicken/Thailand/NP-172/2006 (H5N1) influenza virus with and without treatment are shown in Figure 4A,B, respectively. Infected mice that received *E. coli* lysate had body weight loss since day 1 p.i. Infected mice of all other groups started to show clinical manifestations at day 2–3 p.i. including loss of appetite and body weights. The body weight loss of mice that received PBS, *E. coli* lysate and control scFv and PEN-scFv groups was continued steadily to ~20% reduction and the animals were all dead at days 8, 9, 11 and 11, p.i., respectively. Moribund mice had respiratory and neurological signs including watery nasal discharge, sneezing, dyspnea, ataxia and seizure. Infected mice treated with scFv21, PEN-scFv21, PAb to M1 (one mouse), PAb to H5N1 and rimantadine had body weight loss of ≤ 5% at day 8 p.i.; thereafter they regained their weights. At day 21 p.i. when the experiments were terminated, % survival of the mice treated with 10 mg/kg body weight of PAb to H5N1, PEN-scFv21, rimantadine, scFv21 and PAb to M1 were 100%, 83%, 63%, 33% and 16%, respectively. The survival rates of the infected mice when treated with 5 mg of PEN-scFv21 and scFv21/kg body weight were reduced to 33% and 16%, respectively, indicating that the protective efficacies of the antibodies were dose-dependent.

### 3.6. Quantification of Viral Titer in the Mouse Lungs

**Figure 5:**
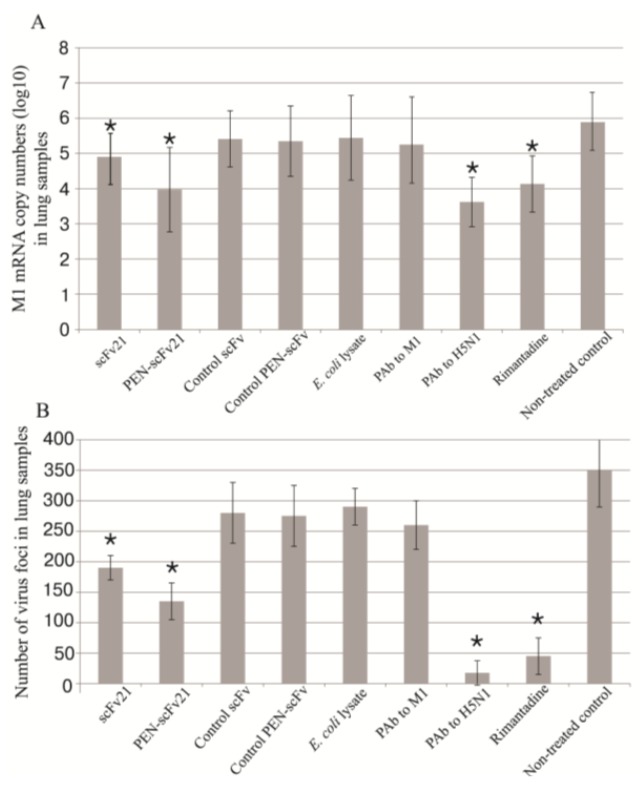
Viral loads in mouse lungs at 96 h post infection with adamantane sensitive A/chicken/Thailand/NP-172/2006 (H5N1) in (**A**) M1 mRNA copy numbers (log_10_) and (**B**) viral plaque numbers in lung samples of all mouse groups. *, significant difference from the non-treated infected control (*p* < 0.05)

Amounts of M1 mRNA in the lungs of the infected mice that received different treatments were quantified by qPCR as shown in Figure 5A, while viral particle numbers were evaluated by viral foci assay (Figure 5B). The infected mice treated with PBS had the most mRNA and viral plaque foci numbers in their lungs. The amounts of the M1 mRNA of the scFv21, PEN-scFv21, PAb to H5N1 and rimantadine groups were different significantly from the PBS treated group (*p* < 0.0001) and the groups treated with control scFv, control PEN-scFv, *E. coli* lysate and PAb to M1 (*p* < 0.05). There was no significant difference in the amounts of the mRNA among the irrelevant scFv, irrelevant PEN-scFv, *E. coli* lysate, PAb to M1 and PBS treated groups (*p* > 0.05). The viral mRNA copy numbers of infected mouse lung samples and viral foci numbers were: PAb to H5N1 < rimantadine < PEN-scFv21 < scFv21 < irrelevant scFv = irrelevant PEN-scFv = *E. coli* lysate = PAb to M1 = culture medium alone.

### 3.7. Comparative Histopathological Features

**Figure 6:**
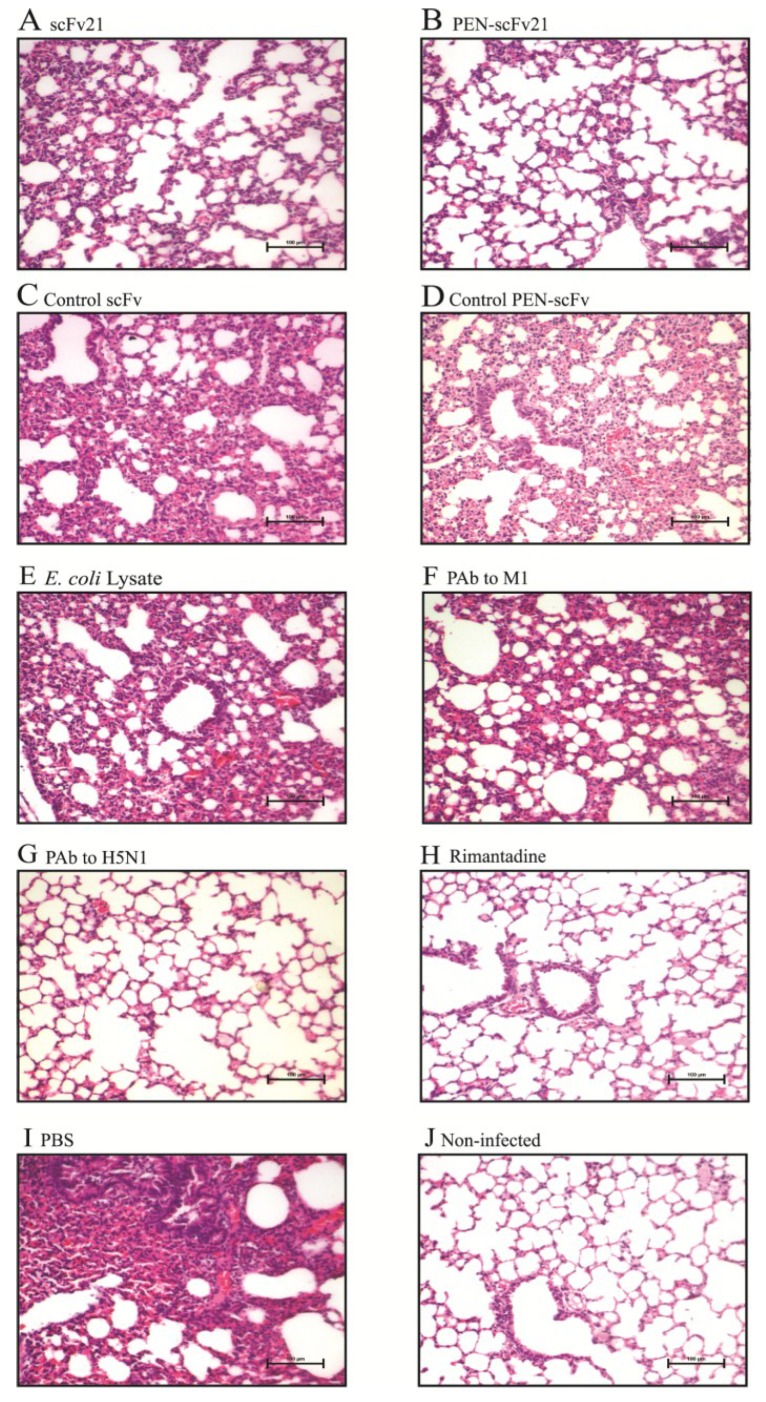
Histopathological features in lung sections of mice infected with 10 MLD_50_ of mouse adapted A/chicken/Thailand/NP-172/2006 (H5N1) at 96 h post infection in comparison with the lung section of a normal mouse. (**A**–**H**) Infected mice treated with 10 mg/kg body weight of scFv21, PEN-scFv21, control scFv, control PEN-scFv, *E. coli* lysate, PAb to M1, PAb to H5N1 and rimantadine; (**I**) Infected mouse treated with PBS and (**J**) normal mouse (H & E; 20× original magnification).

Lung histopathological features of the infected mice that received different treatments at 96 h p.i., in comparison with the histology of the lung of a normal mouse are shown in Figure 6. The untreated infected mice (PBS control group) showed the most severe pulmonary lesion including multifocal necrotizing lymphocytic-neutrophilic interstitial pneumonia and necrosis of the lung tissue. Cellular debris (admixed desquamated epithelial cells and degenerative neutrophils) was found in the bronchiolar and alveolar spaces (Figure 6I). The increasing order of the pathological degrees in the lungs of the treated infected mice were PAb to H5N1 (Figure 6G) = rimantadine (Figure 6H) < PEN-scFv21 (Figure 6B) < scFv21 (Figure 6A) < control scFv (Figure 6C) = control PEN-scFv (Figure 6D) = *E. coli* lysate (Figure 6E) = PAb to M1 (Figure 6F).

### 3.8. Amounts of Virus Antigen in Mouse Lungs

**Figure 7:**
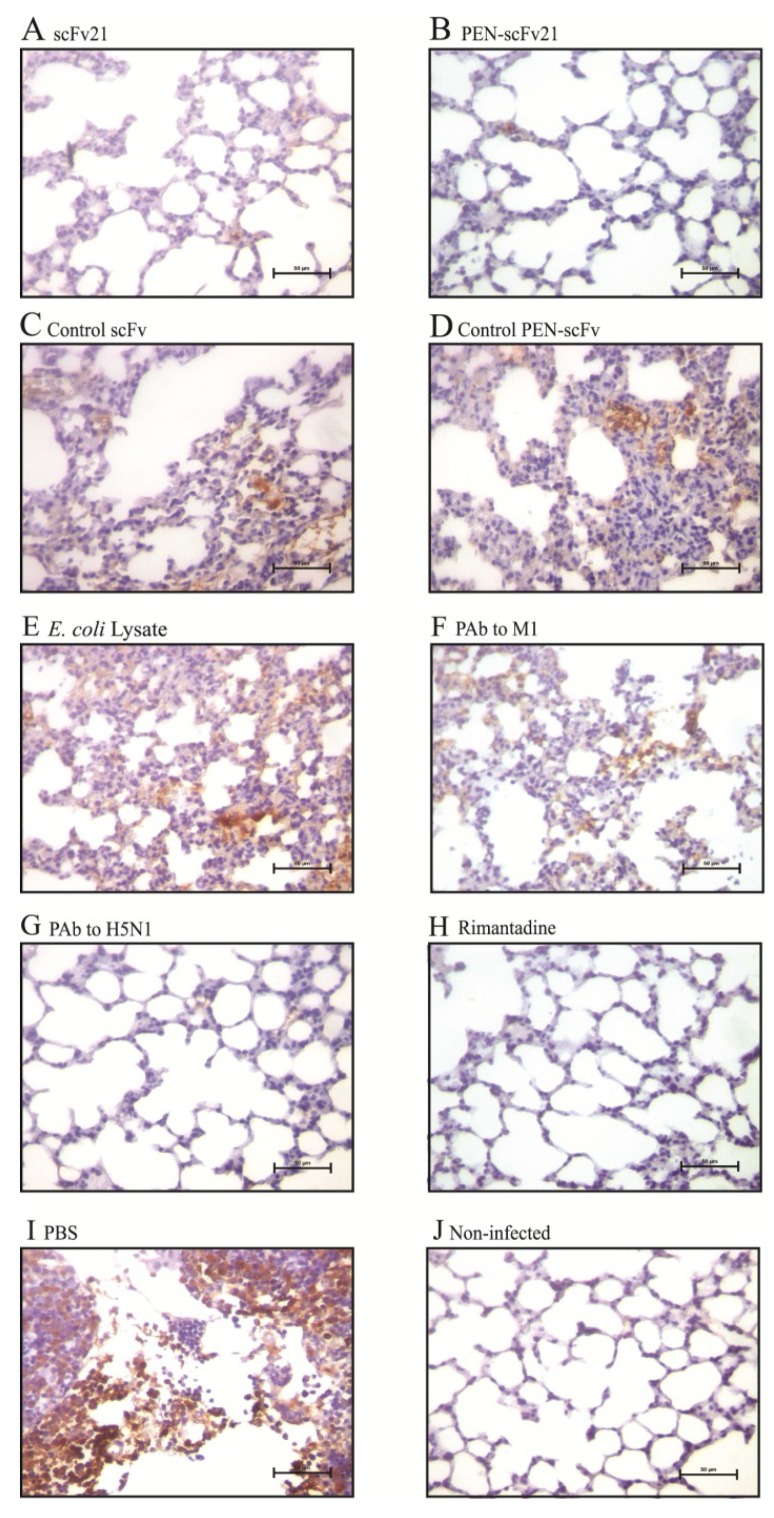
Immunohistochemical staining for influenza virus nucleoprotein (brownish gold pigments) in lung sections (40× original magnification) of mice infected with 10 MLD_50_ of mouse adapted A/chicken/Thailand/NP-172/2006 (H5N1), clade 2, subclade 3 at 96 h post infection. (**A**–**H**) Infected mice treated with 10 mg/kg body weight of scFv21, PEN-scFv21, control scFv, control PEN-scFv, *E. coli* lysate, PAb to M1, PAb to H5N1 and rimantadine; (**I**) Infected mouse treated with PBS and (**J**) normal mouse.

Lung sections of mice stained for the influenza virus nucleoprotein (NP) are shown in Figure 7. Negligible amount of the NP was observed in lung sections of infected mice treated with PAb to H5N1 (Figure 7G) and rimantadine (Figure 7H) in comparison with the sections of the PBS treated infected mice (Figure 7I) which revealed the most of the NP among all infected mouse groups. There was much less NP in the lung sections of mice treated with PEN-scFv21 (Figure 7B) and scFv21 (Figure 7A) compared with the PBS group. Although the amounts of NP in lungs of the groups treated with control scFv (Figure 7C), control PEN-scFv (Figure 7D), *E. coli* lysate (Figure 7E) and PAb to M1 (Figure 7F) were slightly less than the PBS group, they were much more than the PAb to H5N1, the rimantadine, the PEN-scFv and the scFv treated groups.

### 3.9. Phage Mimotopic Peptides that Bound to MD Specific-scFv and Presumptive scFv Epitope

**Figure 8:**
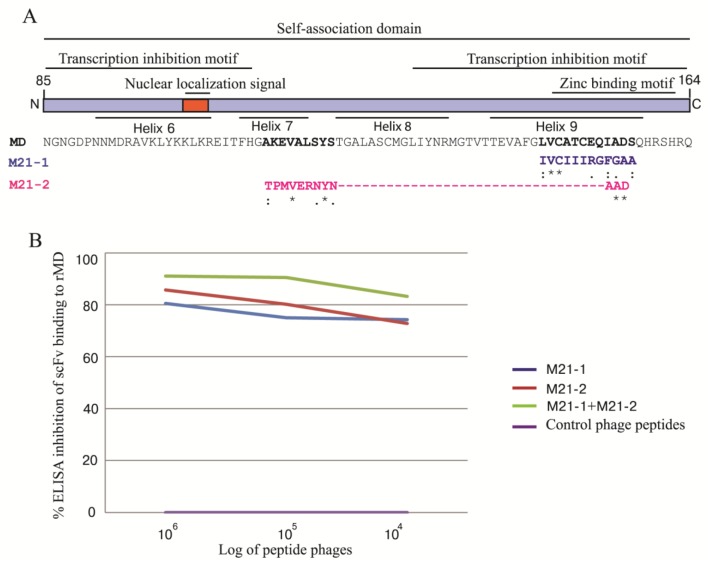
Alignment of the phage mimotopic peptides with the linear sequence of the influenza A virus M1 MD for determining the tentative MD residues bound by the scFv21 and the results of the ELISA inhibition for verification of the phage mimotopes. (**A**) The phage mimotopic peptides, M21-1 and M21-2, matched with residues 146LVCATCEQIADS157 and 112AKEVALSYS120 and 154IAD156 of the M1 MD sequence, respectively; (**B**) ELISA inhibition indicated that the M21-1 and M21-2 phage mimotopes and their mixture blocked the scFv21 binding to the rMD protein which validated the scFv epitope predicted by means of the mimotope search.

Ph.D. 12™ phage display peptide library was used to determine the phage peptides that bound to the MD specific-scFv. The phage clones displaying 12 mer peptides that bound to the scFv21 could be classified into two mimotope (M) types, *i.e.*, M21-1 (IVCIIIRGFGAA) and M21-2 (TPMVERNYNAAD). Individual mimotope sequences were multiply aligned with the influenza virus MD linear sequence (accession no. AY626114) in order to locate residues on MD bound by the scFv21 (presumptive scFv epitope). It was found that the M21-1 matched with residues 146LVCATCEQIADS157 of helix 9 and the M21-2 matched with 112AKEVALSYS120 of helix 7 and 154IAD156 of helix 9 of the MD (Figure 8A). Results of competitive ELISA for determining the ability of the M21-1 and M21-2 mimotopes or their mixture in inhibiting the scFv21 binding to the rMD in comparison with the control mimotopic phages are shown in Figure 8B. Phages carrying the scFv21 mimotopes reduced the scFv binding to the rMD in a dose-dependent manner while the control mimotopic phages did not. 

### 3.10. Homology Modeling and Molecular Docking

**Figure 9:**
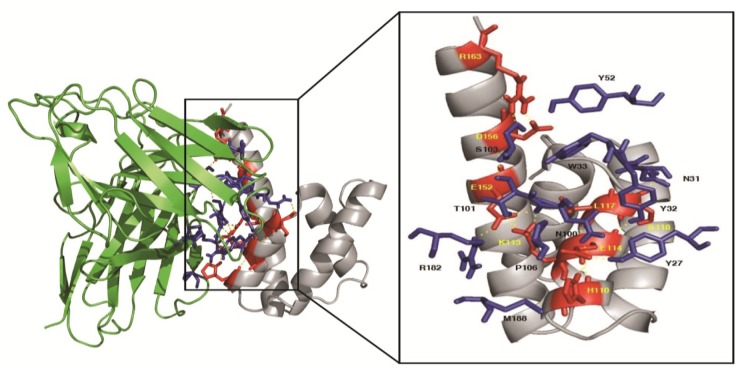
Illustrations of the computerized binding between M1 middle domain and human scFv21. The scFv bound with the least local energy to the residues of helix 7, connecting sequence between helices 7 and 8 and helix 9 of the target protein. Details of the interactive residues between the two parties are shown in Table 2.

The estimated accuracy of the modeled scFv21 and M1 MD are shown in Table 1. The largest cluster size with a minimal local energy for the scFv21 and the M1 MD complex was −242.6 kcal/mol (center energy = −203.2 kcal/mol). According to the docking output, the scFv21 was found to interact with the amino acids located between helices 7–9 of the M1 MD. Table 2 and Figure 9 give details of the interaction between the two parties. The scFv21 used all CDRs and FR2 and FR3 to bind to amino residues of helix 7 (H110, K113, E114 and L117), S118 of the connecting sequence between helices 7 and 8, and E152, D156 and R163 of helix 9 of the MD.

**Table 1:** Estimated accuracy of the scFv21 and M1 MD models.

Protein Name	C-Score	TM-Score	RMSD (Å)	No. of Decoys	Cluster Density
scFv21	0.73	0.81 ± 0.09	4.2 ± 2.8	10165	0.5630
M1 MD	1.43	0.91 ± 0.06	1.0 ± 1.0	10200	1.1111

**Table 2:** Residues of the influenza A virus M1 middle domain bound by the residues and domains of the human scFv21.

Influenza A M1 Middle Domain		Human scFv21		Intermolecular Bond
Amino Acid	Motif	Amino Acid	Domain	
H110	Helix 7	P106	VH-CDR3	Hydrophobic
H110	Helix 7	M188	VL-FR3	Hydrophobic
K113	Helix 7	N100	VH-CDR3	Hydrogen
K113	Helix 7	T101	VH-CDR3	Hydrogen
E114	Helix 7	Y27	VH-CDR1	Hydrogen
E114	Helix 7	Y32	VH-CDR1	Hydrogen
E114	Helix 7	N100	VH-CDR3	Hydrogen
L117	Helix 7	W33	VH-CDR1	Hydrophobic
S118	Between helices 7 and 8	N31	VH-CDR1	Hydrogen
E152	Helix 9	T101	VH-CDR3	Hydrogen
E152	Helix 9	R182	VL-FR2	Salt-bridge
D156	Helix 9	Y52	VH-CDR2	Hydrogen
D156	Helix 9	Y52	VH-CDR2	Hydrogen
R163	Helix 9	S103	VH-CDR3	Hydrogen

## 4. Discussion

A continuous emergence of drug resistant influenza virus strains emphasizes the need of the new anti-influenza remedies. Instead of the small molecular drugs, the strategy that we are proposing for the influenza treatment consists of a combination of ready to use human small antibody fragments, *i.e.*, human scFvs, specific to multiple epitopes of the influenza virus pivotal proteins especially those conserved across the virus subtypes. The human scFvs should be safe (do not induce anti-isotype response in the recipients and do not cause additional inflammation due to Fc deprivation) and in their cell penetrable format, should be accessible to the respective intracellular targets. Normally, each specific antibody molecule uses several residues of the complementarity determining regions (CDRs) as well as the immunoglobulin framework regions (FRs) of both VH and VL domains in binding to the target [40,41]. Therefore the antibodies should cope better with the virus antigenic mutations than the small pharmaceuticals. Recently, human scFvs specific to non-structural protein-1 (NS1) and ion channel protein (M2) that interfered with the respective protein functions and inhibited the virus replication have been reported [29,42]. In this study, cell penetrable human scFv (transbody) that bound specifically to both native M1 and recombinant M1 middle domain was successfully produced *in vitro* using phage display technology. The middle domain (MD) of the influenza virus M1 protein was chosen as the target of the transbody as this domain is not only conserved across the virus subtypes, but also indispensible for the virus infection cycle. Thus, interference with the MD functions by the specific transbody should lead to an abortion of the virus cycle and a consequent reduction of the virus load. 

The MD specific human PEN-scFv produced in this study readily entered the mammalian cells as demonstrated by the laser sectional confocal microscopy of the PEN-scFv treated cells. When used to treat the MDCK cells infected with heterologous H5N1, *i.e.*, adamantine sensitive mouse adapted avian H5N1 (clade 2, subclade 3), the transbody reduced markedly the amounts of the viral mRNA inside the infected cells and in the cell culture fluids in comparison with the infected cells treated with PBS, *E. coli* lysate, control scFv or control PEN-scFv. Moreover, the MD specific-scFv which devoid of the cell penetrable peptide (penetratin, PEN) inhibited also the virus replication (albeit less effective than the PEN-scFv) which should be due to the increased membrane permeability of the infected cells that allowed accessibility of the small antibody to the intracellular target [43,44,45]. In the *in vitro* assay, the PAb to H5N1 (a pool of serum samples of mice recovered from sublethal H5N1 infection), mouse PAb to recombinant M1 and rimantadine were used as anti-virus controls. The infected cells treated with rimantadine and the PAb to H5N1 had the least amounts of the virus mRNA intracellularly and in the culture fluids. The rimantadine is known to inhibit the influenza virus replication by interfering with the M2 ion channel activity which consequently prevented the virus uncoating process [46,47]. The mouse PAb to H5N1 should contain antibodies to a variety of the H5N1 proteins. The most likely mechanisms of the virus replication inhibition by the PAb (each molecule contained intact four-immunoglobulin chains; two Fab and one Fc fragments) should be by interfering with the virus cellular entry mediated by antibodies to hemagglutinin (HA), preventing virus spread by anti-neuraminidase (anti-NA) and blocking the ion channel activity by the antibody to the M2 ectodomain (anti-M2e). The virus entry into cells might be hindered by the cooperative activity of the anti- HA, -NA and -M2e [48]. Moreover, antibodies to the viral surface exposed proteins might stimulate complement system leading to lysis and/or enhanced phagocytosis of the virus particles. The PAb to recombinant M1 did not show any inhibitory activity on the influenza virus replication indicating inaccessibility of the intact antibody to the intracellular target. The inability of the *E. coli* lysate to inhibit the virus replication in comparison with the PBS treated infected cells indicated that the *E. coli* components that might be contaminated in the PEN-scFv/scFv preparations did not contribute to the virus replication inhibition mediated by the PEN-scFv/scFv.

Mice infected with the adamantane sensitive mouse adapted avian H5N1 (clade 2, subclade 3) that received PBS, *E. coli* lysate, irrelevant scFv and irrelevant PEN-scFv had severe morbidity (steady body weight loss) and were all dead by days 7–11 p.i. when the weight loss was ~ 20%. Mice treated with five doses of 5 and 10 mg/kg body weight had 67 and 83% survival, respectively, indicating that the protection afforded by the MD specific antibody fragments was dose-dependent. The protection rate of the infected mice treated with 10 mg/kg body weight of rimantadine was 67% which, more or less conformed to the data reported previously that the drug at 7.5 mg/kg body weight offered 43%–87% protection rates against 10 MLD_50_ of H3N2 human influenza A virus [49]. Surprisingly, the PAb to M1 which were not cell penetrable conferred 16.6% survival of the treated infected mice in three reproducible experiments. Previous study has demonstrated that damaged infected host cells at the late state of influenza virus infection released non-assembled M1 which interacted with the complement C1qA protein and eventually caused high viral propagation in the infected mouse lungs [50]. The PAb against M1 might help to decrease this viral beneficial mechanism and provided the so-observed minimal protection to the infected mice.

Previous study has shown that the virus titer in the infected mouse lung peaked at day 4 p.i. [51]; thus in this study the infected mice that received different treatments were sacrificed for lung histopathology and influenza virus antigen (NP) detection on the fourth day after the virus challenge. The results of both lung histological study and the amounts of the NP revealed similar trend to the virus recovery from the infected cells and the mouse protection tests. The infected mice that received the PBS treatment had the most intense pneumonitis and abundant virus antigen followed by the mice treated with *E. coli* lysate, irrelevant scFv, irrelevant PEN-scFv and PAb to M1 while the mice treated with PAb to H5N1, PEN-scFv and rimantadine showed modest lung inflammation with negligible viral antigen. The scFv treated infected mice had moderate lung lesion and minimal NP antigen.

Although the principles of the phage mimotope search (multiple alignments of the phage mimotopic peptides with the MD linear sequence) and the computerized molecular docking between the 3D structures of the scFv and the MD for identification of the MD region bound by the specific scFv were different, the results of both methods were, more or less, conformed. The scFv21 was found to bind to the conformational epitope formed by residues of helices 7 and 9 which might be juxtaposed upon the protein folding. The MD domain which contained four M1 helices, *i.e.*, 6–9 (residues 85–164 of M1) is known to mediate self-oligomerization useful for the virus assembly and budding [20]. Besides, the MD helix 6 contains one transcriptional inhibition motif (residues 90–109 of M1) and nuclear localization signal sequence (amino acids 101–105 of M1) important for nuclear export of the newly synthesized viral ribonucleoprotein (vRNP) into the cytoplasm for further assembly and budding [52,53,54]. The precise roles of helices 7 and 8 are currently unknown. The helix 9 contained another transcriptional inhibition motif (amino acids 129–164 of M1) and the zinc binding region (148CATCEQIADSQHRSH162) [54,55]. The zinc-bound M1 (residues 148–162 of M1) was thought to play role in virus uncoating by changing the conformation of the M1 under the acidic pH of the virion interior and involved in lethal pathogenicity in mice [55,56]. The scFv21/PEN-scFv21 bound to residues 112AKEVALSYS120 of the helix 7 and 146LVCATCEQIADS157 of the helix 9 and consequently blocked the virus replication. Thus, the scFv/PEN-scFv might mediate the replication inhibition by blocking the zinc-binding region of the M1 and inhibited the virus uncoating as well as inhibiting the MD mediated-self-oligomerization which consequently interfered with the virus assembly and budding. The MD specific-scFv, especially in the cell penetrating format produced in this study has high potential for testing further as a part of, if not a sole, novel anti-influenza remedy.
